# *In vivo *consequences of deleting EGF repeats 8–12 including the ligand binding domain of mouse Notch1

**DOI:** 10.1186/1471-213X-8-48

**Published:** 2008-04-29

**Authors:** Changhui Ge, Tongyi Liu, Xinghua Hou, Pamela Stanley

**Affiliations:** 1 Department of Cell Biology, Albert Einstein College of Medicine, New York, NY-10461, USA; 2 Current Address: Beijing Institute of Radiation Medicine, Beijing, 100850, PR China; 3 Current Address: Chengdu Jingyuan Bio-Science & Technology Co., Ltd. Chengdu, PR China

## Abstract

**Background:**

Notch signaling is highly conserved in the metazoa and is critical for many cell fate decisions. Notch activation occurs following ligand binding to Notch extracellular domain. *In vitro *binding assays have identified epidermal growth factor (EGF) repeats 11 and 12 as the ligand binding domain of Drosophila Notch. Here we show that an internal deletion in mouse Notch1 of EGF repeats 8–12, including the putative ligand binding domain (lbd), is an inactivating mutation *in vivo*. We also show that maternal and zygotic *Notch1*^*lbd*/*lbd *^mutant embryos develop through gastrulation to mid-gestation.

**Results:**

*Notch1*^*lbd*/*lbd *^embryos died at mid-gestation with a phenotype indistinguishable from *Notch1 *null mutants. In embryonic stem (ES) cells, Notch1^lbd ^was expressed on the cell surface at levels equivalent to wild type Notch1, but Delta1 binding was reduced to the same level as in *Notch1 *null cells. In an ES cell co-culture assay, Notch signaling induced by Jagged1 or Delta1 was reduced to a similar level in *Notch1*^*lbd*^and *Notch1 *null cells. However, the *Notch1*^*lbd*/*lbd *^allele was expressed similarly to wild type Notch1 in *Notch1*^*lbd*/*lbd *^ES cells and embryos at E8.75, indicating that Notch1 signaling is not essential for the *Notch1 *gene to be expressed. In addition, maternal and zygotic *Notch1 *mutant blastocysts developed through gastrulation.

**Conclusion:**

Mouse Notch1 lacking the ligand binding domain is expressed at the cell surface but does not signal in response to the canonical Notch ligands Delta1 and Jagged1. Homozygous *Notch1*^*lbd*/*lbd *^mutant embryos die at ~E10 similar to *Notch1 *null embryos. While Notch1 is expressed in oocytes and blastocysts, Notch1 signaling via canonical ligands is dispensable during oogenesis, blastogenesis, implantation and gastrulation.

## Background

Notch1 is a heterodimeric, type I transmembrane receptor that is required for cell fate decisions throughout the metazoa [1,2]. The Notch1 extracellular domain contains 36 tandem epidermal growth factor-like (EGF) repeats, and three Lin/Notch repeats. Of the 36 EGF repeats in Drosophila Notch, deletion of only EGF repeats 11 and 12 prohibits the binding of the Notch ligands Delta and Serrate in *in vitro *binding assays [3,4]. Notch signaling in mammals is also initiated by binding to canonical Notch ligands (Delta and Jagged) on adjacent cells. Ligand binding activates Notch signaling through two proteolytic cleavage events, first in the extracellular domain by the ADAM10 metalloprotease [5], and subsequently in the transmembrane domain by a presenilin complex with γ-secretase activity [6,7]. The released Notch intracellular domain (NICD) translocates to the nucleus and binds to the CSL (CBF1, Suppressor of hairless, Lag-1) transcriptional repressor [6]. The NICD/CSL complex recruits co-activators including mastermind (MAML), and up-regulates a number of target genes including the HES (Hairy/Enhancer of Split) family of basic helix-loop-helix transcriptional regulators.

The *Notch1 *gene has been inactivated in mice by inserting a neomycin gene into EGF32 (*Notch1*^*in*32^; [8]) or by deleting a large internal fragment from aa 1056–2049 that spans the transmembrane domain (*Notch1*^*tm*1/*Con*/1^; [9]). The *Notch1*^*in*32 ^mutation generates a null allele [10] and *Notch1*^*tm*1/*Con*/1 ^homozygotes have an indistinguishable embryonic lethal phenotype. *Notch1 *null embryos die at mid-gestation around E10, with severe defects in somitogenesis, neurogenesis, vasculogenesis and cardiogenesis. The phenotype of flies expressing Notch with the ligand binding domain deletion is not known. Thus in order to investigate biological consequences of this type of Notch mutation, we generated a mouse *Notch1 *mutation termed *Notch1*^*lbd *^by deleting EGF repeats 8–12 (aa 290–481), which include the putative Notch1 ligand binding domain. We show that Notch1^lbd ^is expressed on the cell surface but cannot bind to canonical Notch ligands nor signal in response to these ligands. Homozygous *Notch1*^*lbd*/*lbd *^embryos exhibit defects during embryogenesis similar to *Notch1 *null mutants. However, Notch1^lbd ^transcripts are expressed at levels similar to wild type in ES cells and in E8.75 *Notch1*^*lbd*/*lbd *^embryos, indicating that canonical Notch1 signaling is not essential for *Notch1 *gene expression during early embryogenesis. In addition, while *Notch1 *is expressed in oocytes and blastocysts [11,12], we show that oocyte-specific inactivation of Notch1 does not affect oogenesis or fertilization and that maternal and zygotic mutants proceed normally through blastogenesis, implantation and gastrulation.

## Results

### Notch signaling defects in *Notch1*^*lbd*/*lbd *^embryos

**Figure 1:**
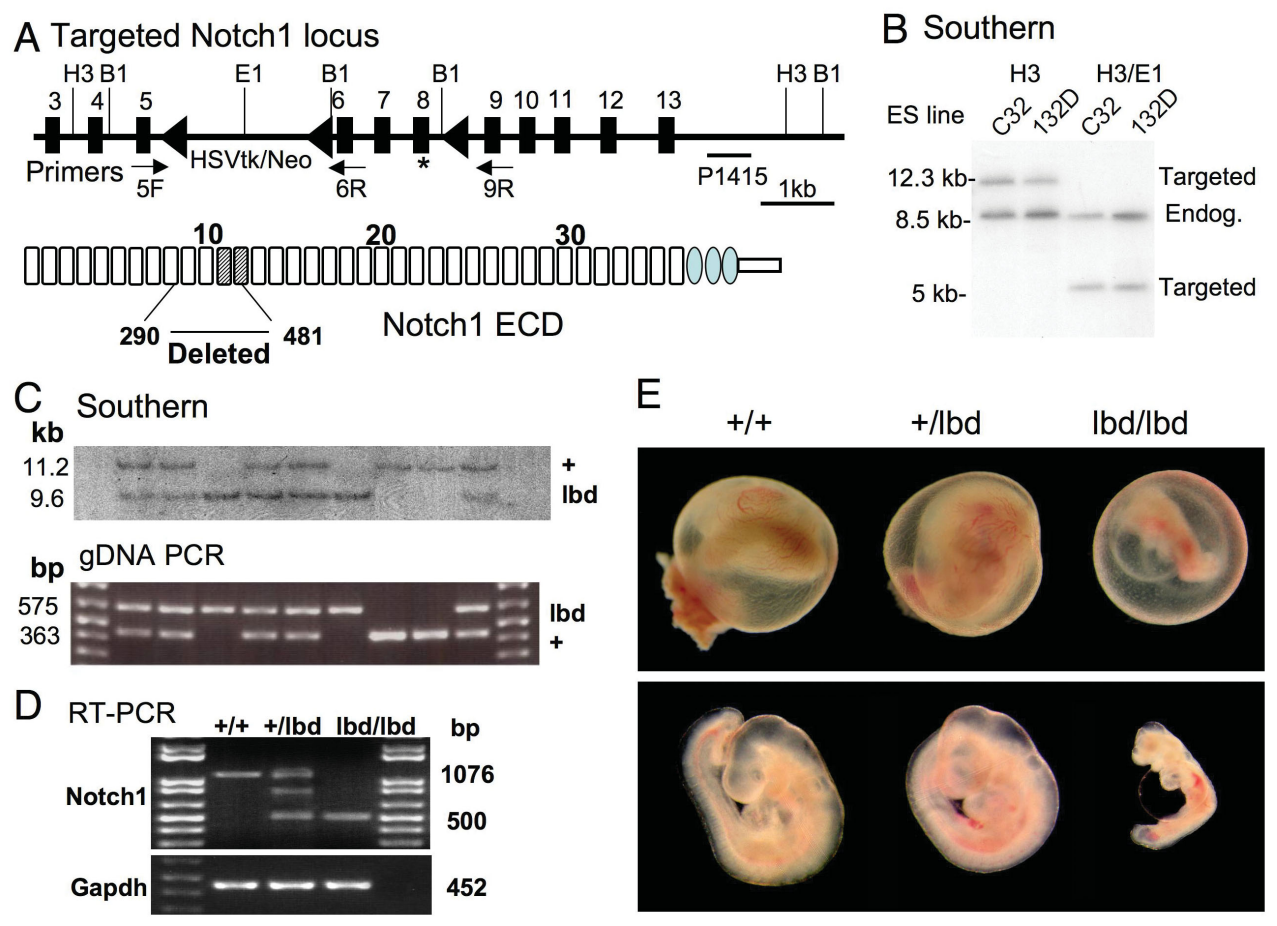
**Targeting of the *Notch1 *gene**. (A) Schematic representation of the floxed region of the mouse *Notch1 *gene. Exons 6 – 8 (* designates a T466A point mutation termed Notch1^12f ^described by [13]) and the HSVTk/Neo cassette were removed by *Cre *recombinase to generate the *Notch1*^*lbd *^allele. The diagram of Notch1 ECD shows EGF repeats as rectangles and LIN repeats as ovals. The ligand binding domain in EGF repeats 11 and 12 is striped. Amino acids 290–481 were removed by the *Notch1*^*lbd *^mutation. PCR primers 5F, 6R and 9R and the P1415 probe are indicated. B1: *BamH*I; E1: *EcoR*I; H3: *Hind*III. (B) Southern blot analysis of two targeted ES clones (C32 and 132D) by hybridization with probe P1415 after digestion with *Hind*III or *Hind*III and *EcoR*I. (C) Southern blot analysis and PCR genotyping of yolk sac genomic DNA from E9.5 embryos from a *Notch1*^+/*lbd *^heterozygous cross. Genomic DNA was digested with *BamH*I and probed with P1415. (D) RT-PCR analysis of total RNA from ES cells of the genotypes shown. A hybrid band was obtained from *Notch1*^+/*lbd *^cDNA. (E) E9.5 embryos exhibited defective vascularization of yolk sac and retarded development of *Notch1*^*lbd*/*lbd *^embryos, but no apparent differences between wild type and heterozygous progeny.

To generate mice with Notch1 lacking the putative ligand binding domain, embryonic stem (ES) cells with *lox*P sequences flanking exons 6 – 8 of mouse *Notch1 *were generated by gene targeting (Fig. 1A; [13]). Exons 7 and 8 encode EGF11 and EGF12 and exon 6 was included in order that the mutant Notch1 was ~20 kDa lower in molecular weight. Two independent ES colonies selected for resistance to G418 were shown by Southern analysis to carry a targeted *Notch1 *allele (Fig. 1B). Chimeric mice carrying the mutant allele were crossed with mice expressing the MeuCre40 recombinase transgene [14] to obtain mice with a *Notch1*^*lbd *^allele after deletion of exons 6 – 8 along with the HSVtk/Neo cassette (Fig. 1A). Southern blot and PCR analysis of genomic DNA were used to genotype E9.5 embryos of *Notch1*^+/*lbd *^crosses (Fig. 1C). All expected genotypes were represented at this stage. However, only wild type and heterozygous pups were born from 6 litters (Table 1).

**Table 1:** Progeny of the cross *Notch1*^+/*lbd *^X *Notch1*^+/*lbd*^.

**No. Litters**	**Pups/Embryos**	**Stage**	**Genotype**		
	
			**+/+**	+/lbd	lbd/lbd
6	35	P10	12	23	0
6	44	E9.5	12	21	11

Pups at postnatal day 10 (P10) were genotyped from litters of 5 *Notch1*^+/*lbd *^females mated to 5 *Notch1*^+/*lbd *^males. Yolk sacs of E9.5 embryos were genotyped following timed matings of 6 *Notch1*^+/*lbd *^females to 4 *Notch1*^+/*lbd *^males.

To determine when *Notch1*^*lbd*/*lbd *^embryos die, embryos from *Notch1*^+/*lbd *^crosses were examined during embryogenesis. *Notch1*^*lbd*/*lbd *^embryos were indistinguishable from wild type at ~E8.75, but by ~E9.5 *Notch1*^*lbd*/*lbd *^embryos were severely growth-retarded, with a tube-like heart, distended pericardial sac, and defective vascularization of the yolk sac (Fig. 1D). By ~E10.5, many mutant embryos were resorbed and all mutant embryos were resorbed by ~E11.5. Therefore *Notch1*^*lbd*/*lbd *^embryos exhibited global defects in Notch signaling with a phenotype indistinguishable from *Notch1*^*in*32 ^[8] or *Notch1*^*tm*1/*Con*/1 ^[9] null embryos.

### Notch1^lbd ^is expressed at the cell surface but does not signal

**Figure 2:**
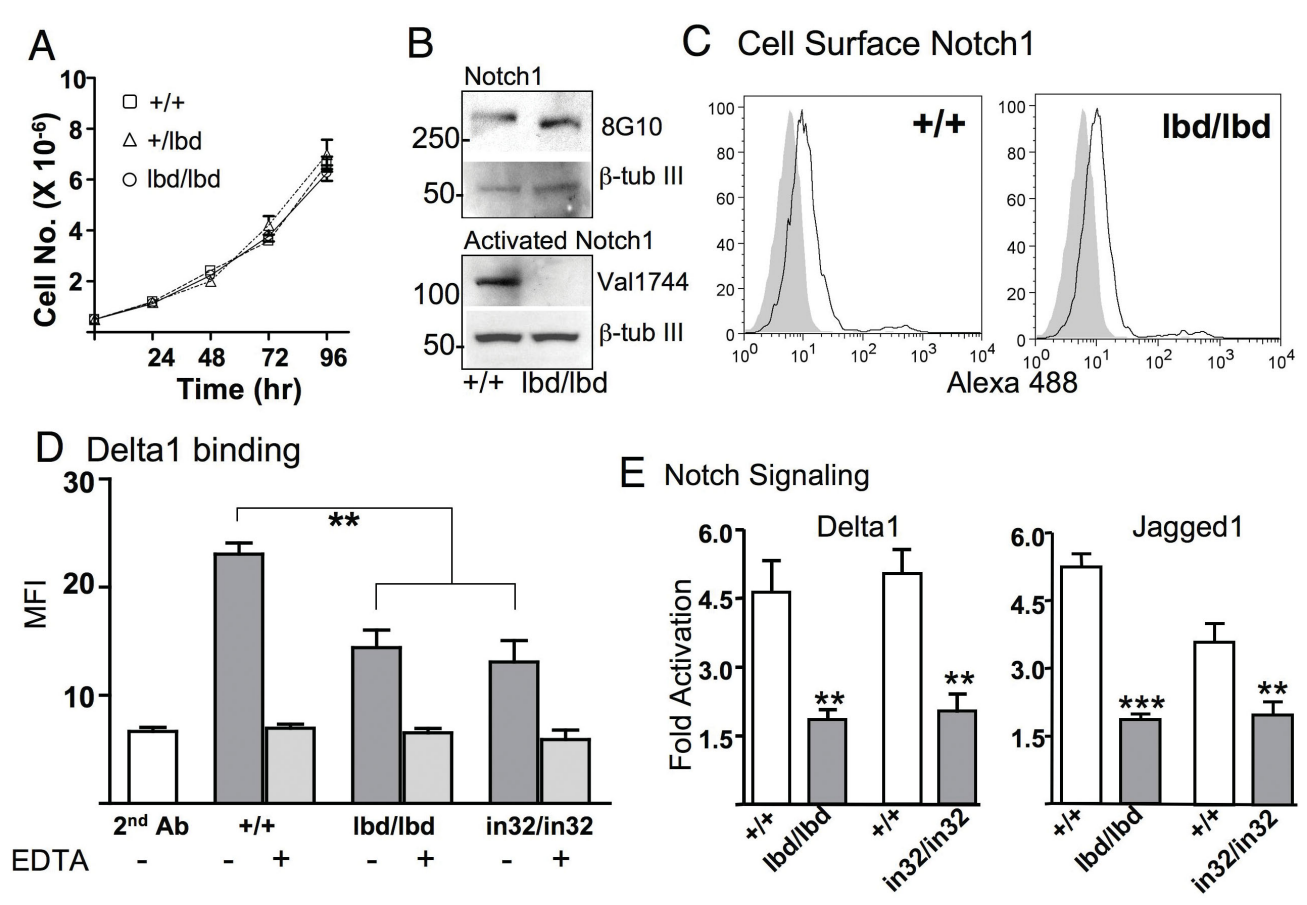
**Notch1 lacking the ligand binding domain is expressed on the cell surface but does not signal**. (A) Growth curves of ES cells isolated from E3.5 *Notch1*^+/+^, *Notch1*^+/*lbd *^and *Notch1*^*lbd*/*lbd *^blastocysts. Bars represent mean ± SD. (B) Western blot analysis of ES cell lysates (50 μg protein). Full length Notch1 was detected by antibody 8G10 and activated Notch1 was detected by antibody Val1744. Blots were stripped and reprobed using anti-β-Tubulin III. Data are representative of 3 experiments. (C) Flow cytometry of cell surface Notch1 in *Notch1*^*lbd*/*lbd *^and *Notch1*^+/+ ^ES cells using anti-Notch1 ECD antibody 8G10 followed by Alexa-488 conjugated anti-hamster IgG. Grey profiles are secondary antibody alone. Profiles are representative of two experiments. (D) Notch ligand binding. ES cells were incubated with soluble Delta1-Fc followed by PE-conjugated anti-human IgG and analyzed by flow cytometry. *Notch1*^*in*32/*in*32 ^null ES cells were line 290-2. EDTA in the binding buffer inhibited binding to all Notch receptors. Bars represent mean ± SEM; n = 5 for *Notch1*^+/+ ^and *Notch1*^*lbd*/*lbd*^; n = 3 for *Notch1*^*in*32/*in*32 ^ES cells. (E) ES cells were assayed for Notch signaling after transfection of the Notch TP-1 reporter construct by co-culturing with L cells expressing Delta1 or Jagged1 compared to control L cells. Bars represent fold-activation ± SEM for *Notch1*^+/+ ^(white), *Notch1*^*lbd*/*lbd *^and *Notch1*^*in*32/*in*32 ^(gray) (n = 4; ** *P *< 0.01, *** *P *< 0.001).

Blastocysts from heterozygous *Notch1*^+/*lbd *^crosses were used to isolate embryonic stem (ES) cell lines of each genotype. Reverse transcription (RT)-PCR of total RNA showed that *Notch1*^*lbd*/*lbd *^ES cells expressed Notch1 transcripts at levels similar to wild type and heterozygous ES cells (Fig. 1E). *Notch1*^*lbd*/*lbd *^ES cells had a similar growth rate to *Notch1*^+/*lbd *^and *Notch1*^+/+ ^ES cells (Fig. 2A). This was also observed with *Notch1 *null ES cells [10]. An antibody to the extracellular domain of Notch1 (8G10) detected the ~300 kD full length Notch1 in wild type ES cells and the ~280 kDa truncated Notch1 in *Notch1*^*lbd*/*lbd *^ES cells (Fig. 2B). The ~180 kDa Notch1 extracellular domain was not routinely observed, but when present it was in similar amounts in *Notch1*^*lbd*/*lbd *^and *Notch1*^+/+^cells. Flow cytometry showed that equivalent amounts of wild type and mutant Notch1 receptors were present on the surface of *Notch1*^+/+ ^and *Notch1*^*lbd*/*lbd *^ES cells, respectively (Fig. 2C). Therefore the internal deletion that includes the putative ligand binding domain did not affect Notch1 stability or trafficking to the cell surface.

When Notch1 binds canonical Notch ligands, it undergoes cleavage by γ-secretase and the new N-terminus of activated Notch1 may be detected by the antibody Val1744 [15]. Western blot analysis revealed a robust signal for activated Notch1 in cultured wild type ES cells but no corresponding signal was observed in *Notch1*^*lbd*/*lbd *^ES cells (Fig. 2B). Thus while Notch1^lbd ^is expressed at the cell surface it is not activated under conditions that activate wild type Notch1, presumably because of the loss of its ligand binding domain. Indeed the Notch ligand Delta1 had reduced binding to *Notch1*^*lbd*/*lbd *^ES cells (Fig. 2D). ES cells express each of the four mammalian Notch receptors and all would be expected to bind Delta1. To confirm that the reduced binding of Delta1 to *Notch1*^*lbd*/*lbd *^is due to the *Notch1*^*lbd *^mutation, we examined Delta1 binding to *Notch1 *null ES cells (*Notch1*^*in*32/*in*32^) termed 290-2 which lack Notch1 on the cell surface [10]. The binding of Delta1 to 290-2 cells was reduced to the same extent as to *Notch1*^*lbd*/*lbd *^cells (Fig. 2D). Therefore, the deletion of EGF repeats 8–12 in mouse Notch1 eliminates Delta1 binding to Notch1 as expected, but binding to other Notch receptors remains. This residual binding was prevented by including EDTA in the binding buffer under conditions that prevent Notch/ligand binding but do not release Notch receptors from the cell surface. Delta1-induced Notch1 signaling was also reduced in *Notch1*^*lbd*/*lbd *^ES cells as shown by a co-culture reporter assay which detects signaling through all four Notch receptors (Fig. 2E). A second canonical Notch ligand, Jagged1, was also defective at inducing Notch signaling in *Notch1*^*lbd*/*lbd *^ES cells (Fig. 2E). Delta1- and Jagged1-induced Notch signaling was also reduced, but not eliminated, in *Notch1*^*in*32/*in*32 ^ES cells which are Notch1 null [10] (Fig. 2E). Residual Notch signaling presumably reflects the presence of the other three Notch receptors.

### Notch1 signaling is not essential for *Notch1 *gene expression in early embryogenesis

**Figure 3:**
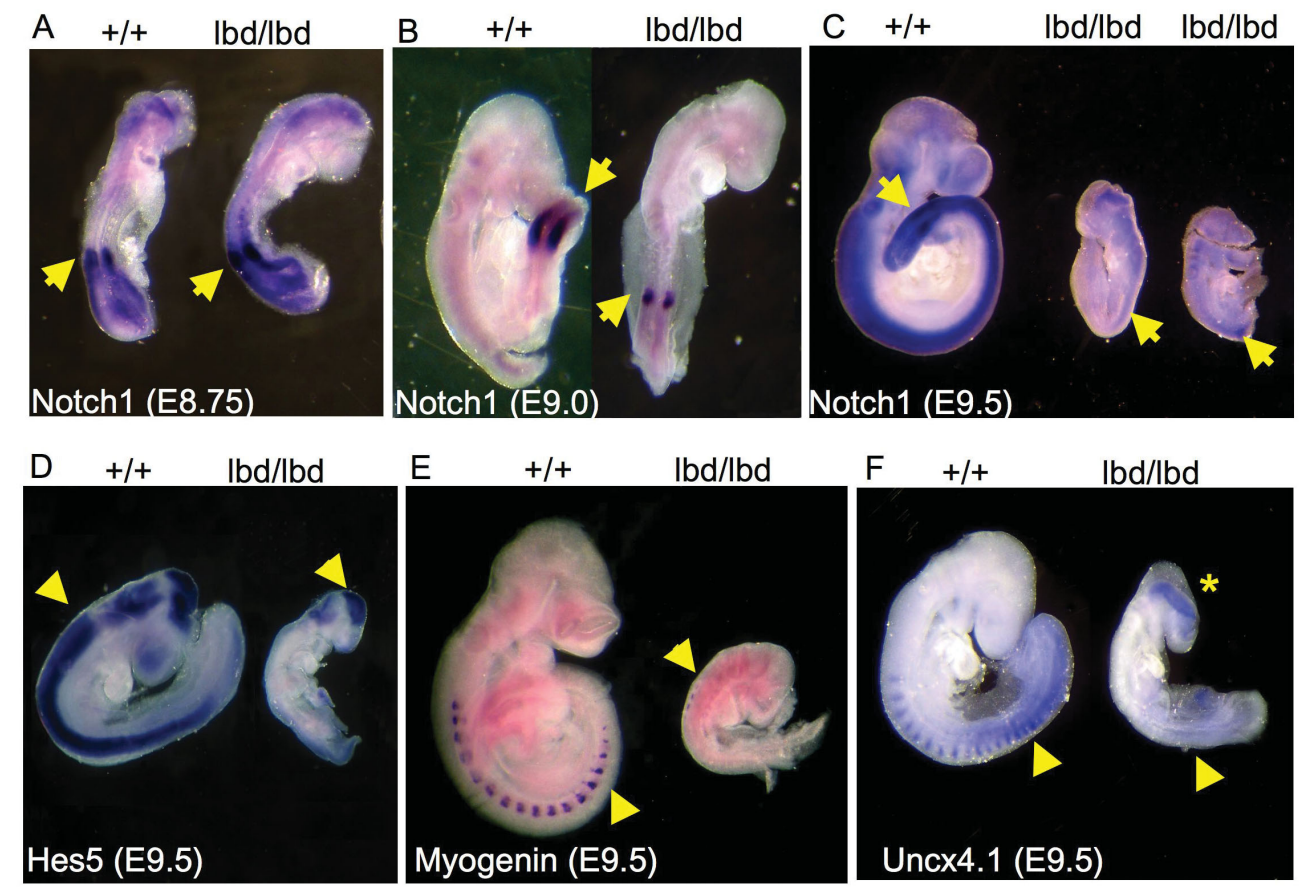
**Whole mount *in situ *hybridization of Notch1 pathway and somitogenic genes**. Control (*Notch1*^+/+^) embryos (left) and mutant *Notch1*^*lbd*/*lbd *^denoted lbd/lbd (right) embryos were probed together. Arrows point to highest expression. (A) *Notch1 *expression in E8.75 control and *Notch1*^*lbd*/*lbd *^embryos was similar (~9.7 kb probe). (B) At E9.0 *Notch1 *expression was reduced in *Notch1*^*lbd*/*lbd *^compared to control embryos (~4.7 kb probe). (C) At E9.5 *Notch1 *expression was barely detectable in *Notch1*^*lbd*/*lbd *^embryos (~9.7 kb probe). (D) Expression of the Notch target gene *Hes5 *was reduced in mutant embryos at E9.5, with residual expression in brain. (E) *Myogenin *was poorly and diffusely expressed in E9.5 *Notch1*^*lbd*/*lbd *^embryos. (F) *Uncx4.1 *was expressed in the caudal compartment of formed somites of control but was missing from the somitic region of E9.5 *Notch1*^*lbd*/*lbd *^embryos. *Uncx4.1 *was up-regulated in the midbrain (asterisk) of E9.5 *Notch1*^*lbd*/*lbd *^embryos (n ≥ 3 for mutant embryos for each probe).

The *Notch1*^*lbd *^mutant allele is transcribed similarly to the wild type allele in *Notch1*^*lbd*/*lbd *^ES cells (Fig. 1E) but *Notch1*^*lbd*/*lbd *^ES cells are defective in Notch1 signaling (Figs. 1 and 2). The *Notch1*^*lbd *^mutant allele therefore allowed us to determine if signaling via *Notch1 *is required for the *Notch1 *gene to be expressed *in vivo*. *Notch1*^+/*lbd *^females were crossed with *Notch1*^+/*lbd *^males and embryos were examined at mid-gestation. *Notch1*^*lbd*/*lbd *^embryos were morphologically indistinguishable from wild type embryos at E8.75 and were examined for *Notch1 *gene expression by *in situ *hybridization. It can be seen in Fig. 3A that Notch1 was expressed in the presomitic mesoderm (PSM) the forming somite (arrows) and the neural tube. The hybridization signal obtained for wild type and *Notch1*^*lbd*/*lbd *^embryos was similar. Therefore expression of the *Notch1 *gene was not markedly altered by the loss of canonical Notch1 signaling in early embryogenesis. By contrast, following removal of Pofut1 [16] or Mind bomb 1 [17,18], both of which inhibit Notch signaling through all four Notch receptors, *Notch1 *expression is reduced in the PSM and enhanced in neural tube and mesencephalon of E8.75 embryos. At E9.0, Notch1 transcripts were markedly reduced in *Notch1*^*lbd*/*lbd *^embryos (Fig. 3B). By E9.5 when *Notch1*^*lbd*/*lbd *^embryos were dying, *Notch1 *gene expression was severely reduced (Fig. 3C).

Whole mount *in situ *hybridization provided additional confirmation that the *Notch1*^*lbd *^mutation inactivates Notch1 signaling. The Notch1 target and somitogenic genes *Hes5, Myogenin *and *Uncx4.1 *were examined in embryos at E9.5. At that stage *Hes5 *is expressed in neural tube, brain and the forming and formed somites. The *Hes5 *gene is a known target of Notch1 signaling and its expression was severely reduced in *Notch1*^*lbd*/*lbd *^embryos (Fig. 3D). *Myogenin *is expressed in mature somites of wild type embryos at E9.5. In *Notch1*^*lbd*/*lbd *^embryos which had 13–17 (n = 3) poorly-formed somites, *Myogenin *expression was greatly reduced (Fig. 3E). The myogenic transcription factor *Uncx4.1 *is expressed on the posterior side of mature somites and in the PSM of wild type embryos at E9.5. In *Notch1*^*lbd*/*lbd *^embryos, expression in somites and PSM was lost (Fig. 3F). However, expression of *Uncx4.1 *was induced in brain in the absence of Notch1 signaling, as observed previously in embryos defective in signaling through all four Notch receptors [16]. We previously showed that cyclin D1 expression is markedly reduced in *Notch1*^*lbd*/*lbd *^embryos [19]. Importantly therefore, the Notch1 signaling defects observed in *Notch1*^*lbd*/*lbd *^embryos are not rescued by non-canonical Notch1 ligands that might bind to the large portion of the Notch1 extracellular domain that remains in Notch1^lbd^.

### Maternal and zygotic *Notchl*^*lbd/lbd *^mutant blastocysts implant and develop through gastrulation

**Figure 4:**
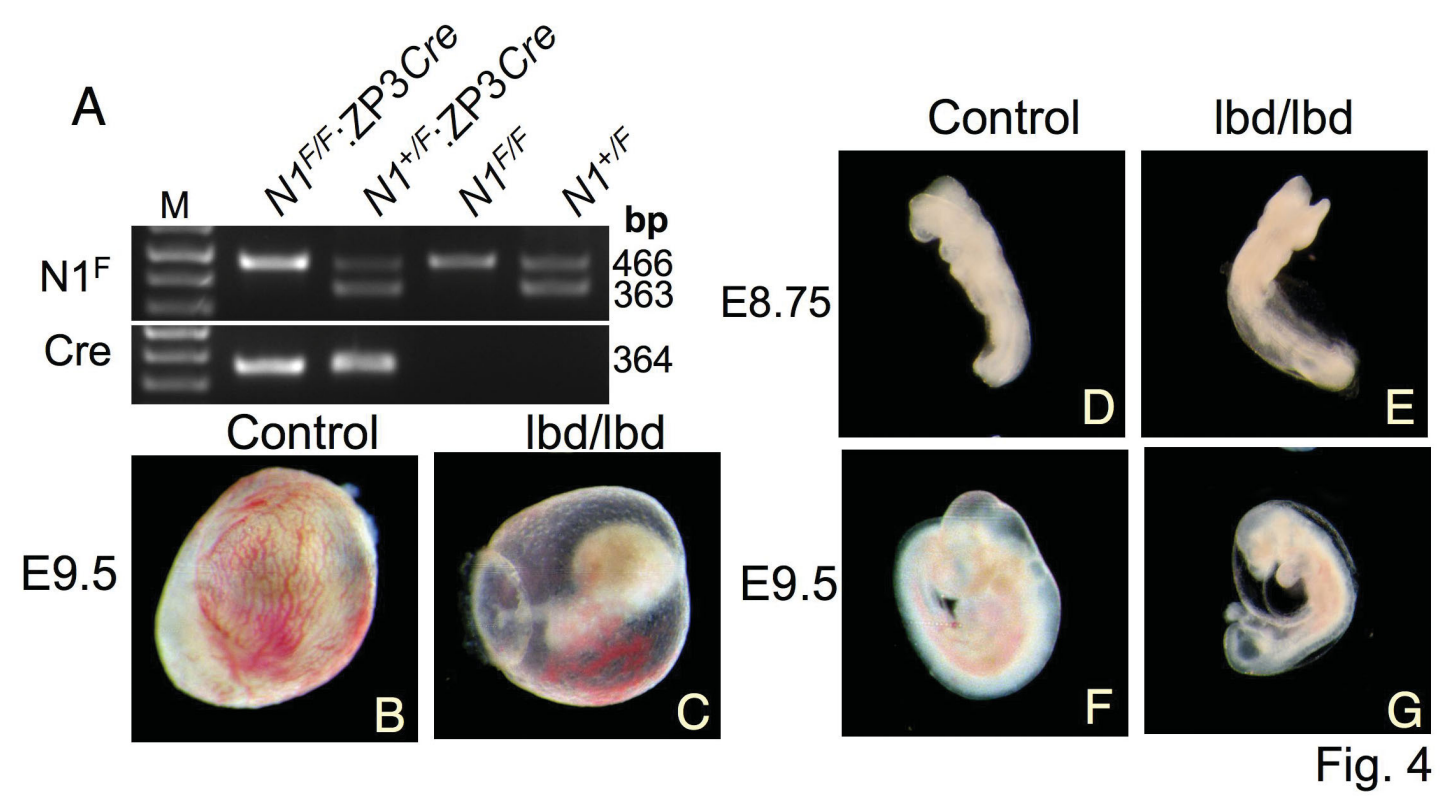
***Notch1*^*lbd*/*lbd *^maternal and zygotic null embryos**. (A) PCR of genomic DNA using primers 5F and 6R (Fig. 1A), and ZP3*Cre *primers. N1^F^: Notch1 floxed allele; Cre: ZP3*Cre *transgene. (B) – (C) E9.5 yolk sac with embryos from crosses between *Notch1*^*F*/*F*^:ZP3*Cre *females and *Notch1*^+/*lbd *^males. (D) – (E) E8.75 *Notch1*^+/*lbd *^and *Notch1*^*lbd*/*lbd *^embryos. (F) – (G) E9.5 *Notch1*^+/*lbd *^and *Notch1*^*lbd*/*lbd *^embryos. Representative results from a total of 14 E8.75 embryos and 29 E9.5 embryos.

Following oocyte-specific deletion of *Pofut1 *or *RBP-Jκ *null oocytes are fertilized and mutant blastocysts develop through gastrulation [20,21]. Pofut1 [16] and RBP-Jκ [22] are essential for Notch signaling through all four Notch receptors. Pofut1 transfers fucose to Notch receptors and RBP-Jκ complexes with the cleaved ICD of all Notch receptors, but both activities might have effects that are independent of the Notch pathway. Thus it was of interest to determine if *Notch1*^*lbd*/*lbd *^blastocysts could develop and implant because Notch1 is expressed in oocytes, fertilized eggs and blastocysts [11,12]. To obtain maternal and zygotic mutant blastocysts, females homozygous for the *Notch1 *floxed allele (Fig. 1A) and carrying a ZP3*Cre *transgene were generated (Fig. 4A). *Notch1*^*F*/*F*^:ZP3*Cre *females were mated with *Notch1*^+/*lbd *^or wild type males. Pups and E9.5 embryos were genotyped (Table 2). At birth, all pups from *Notch1*^*F*/*F*^:ZP3*Cre *by wild type crosses were heterozygous showing that the Cre recombinase was highly efficient since the *Notch1*^*F *^allele was not transmitted. At E9.5, 29 embryos from 4 crosses included 15 mutants (*Notch1*^*lbd*/*lbd*^) and 14 heterozygotes (*Notch1*^+/*lbd*^). Therefore, eggs with Notch1 lacking the ligand binding domain were fertilized by sperm that also lacked functional Notch1 and gave the same number of E9.5 embryos as eggs fertilized with a *Notch1*^+ ^sperm.

**Table 2:** Progeny of crosses *N1*^*F*/*F*^*:ZP3Cre *X *N1*^+/+ ^and *N1*^*F*/*F*^*:ZP3Cre *X *N1*^+/*lbd*^.

**Female**	**Male**	**No. Litters**	**Pups/Embryos**	**Stage**	**Genotype**		
	
					**+/+**	+/lbd	lbd/lbd
N1^F/F^:ZP3Cre	N1^+/+^	7	42	P10	0	42	0
N1^+/*F*^:ZP3Cre	N1^+/+^	6	40	P10	21	19	0
N1^F/F^:ZP3Cre	N1^+/*lbd*^	3	10	P10	0	10	0
N1^F/F^:ZP3Cre	N1^+/*lbd*^	4	29	E9.5	0	14	15

Pups at postnatal day 10 (P10) were genotyped from litters of 8 *Notch1*^*F*/*F*^:ZP3Cre *or 5 Notch1*^+/*F*^:ZP3Cre females mated to 6 *Notch1*^+/+ ^or 2 *Notch1*^+/*lbd *^males. Yolk sacs of E9.5 embryos were genotyped following timed matings of 4 *Notch1*^*F*/*F*^:ZP3Cre females to 3 *Notch1*^+/*lbd *^males. N1, Notch1

Mutant embryos derived from *Notch1*^*lbd*/*lbd *^eggs and therefore lacking maternal and zygotic transcripts of functional Notch1 were examined at E8.75 and E9.5. All *Notch1*^*lbd*/*lbd *^E9.5 embryos were surrounded by a yolk sac with defective vascularization (Figs. 4B, C). *Notch1*^+/*lbd *^and *Notch1*^*lbd*/*lbd *^embryos at E8.75 (8 vs. 6 from 2 litters) were morphologically indistinguishable (Figs. 4D, E). By E9.5, the *Notch1*^*lbd*/*lbd *^embryos were significantly smaller than controls, and the Notch1 null mutant phenotype was readily apparent (Figs. 4F, G). All developmental defects in *Notch1*^*lbd*/*lbd *^embryos arising from mutant blastocysts with or without maternal Notch1 transcripts appeared similar. In addition, mutant embryos were morphologically similar to controls at E8.75 (Fig. 4D, E). Therefore Notch1 signaling induced by canonical Notch ligands is not required for oogenesis, ovulation, fertilization or any of the developmental steps involved in blastogenesis, implantation or gastrulation.

## Discussion

The *Notch1*^*lbd *^mutation is a novel Notch1 inactivating mutation that gives a null phenotype with a defective Notch1 that is expressed at normal levels at the cell surface. The Notch1 cleavage site mutant which is not efficiently cleaved by γ-secretase and is defective in Notch1 signaling [15] may also be expressed at the cell surface but this point has not been directly investigated. However, treatment of T cells with inhibitors of γ-secretase causes an accumulation of a membrane-bound Notch1 stable intermediate [23]. In Notch1^lbd^, removal of aa 290–481 from the Notch1 ECD does not inhibit Notch1 synthesis, trafficking or stability at the plasma membrane. Nevertheless, Notch1^lbd ^exhibits reduced signaling through Delta1 and Jagged1 to the same extent as *Notch1 *null ES cells in a co-culture reporter assay. Moreover, the binding of Delta1 is reduced to the same extent as in cells that lack Notch1 altogether. This is consistent with *in vitro *binding experiments which identify EGF repeats 11 and 12 of Drosophila Notch as necessary for canonical ligand binding *in vitro *[3,4]. However, the Drosophila Notch EGF11/12 deletion mutant has not been investigated for Notch signaling abilities, either in co-culture assays or,*in vivo *in the fly.

*Notch1*^*lbd*/*lbd *^embryos die at ~E10 with the same developmental phenotype as embryos that lack Notch1 [8,9]. It is of interest that, despite the cell surface expression of most of the Notch1 extracellular domain in Notch1^lbd^, there appear to be no non-canonical Notch ligands that rescue mutant embryos at mid-gestation. Also of interest, is the fact that heterozygotes develop similarly to wild type mice, and thus Notch1^lbd ^does not behave in a dominant negative fashion even though it is expressed at the cell surface in similar amounts to wild type Notch1. It will be of interest to see if *Notch1*^+/*lbd *^heterozygotes exhibit the more subtle Notch1 signaling defects observed in the inner ear [24], and in cell competition experiments [25] with other *Notch1*^+/*null *^heterozygotes.

Whole mount *in situ *hybridization showed that *Notch1*^*lbd*/*lbd *^embryos exhibit markedly reduced expression of the Notch1 target genes *Hes5 *and *cyclin D1 *[19] and the somitogenic genes *Myogenin *and *Uncx4.1*. Similar results were observed in Notch1 null mutants [8,9]. However, at E8.75 Notch1 transcripts were expressed at similar levels in mutant and wild type controls (Fig. 3A). Previous studies have shown that removal of Notch1 delays somitogenesis at the 3-5 somite stage around E8 showing that Notch1 signaling is active at this stage, even though there are no apparent changes in size, overall appearance, neurogenesis or cell death at E8 [9]. The embryos in Fig. 3A show that *Notch1 *gene expression in early embryogenesis is not solely controled by Notch1 signaling. This conclusion can also be drawn from the equivalent expression of Notch1 transcripts in *Notch1*^*lbd*/*lbd *^and *Notch1*^+/+ ^ES cells (Fig. 1D). However, by E9.0 *Notch1*^*lbd*/*lbd *^embryos had markedly less *Notch1 *expression than controls (Fig. 2E). This may suggest that Notch1 signaling and *Notch1 *gene expression operate in a feedback loop at this stage as suggested from results of overexpression of Notch1 ICD in T cells [26,27] or C2C12 cells [28]. However, overexpression experiments may induce abberrant regulation of the *Notch1 *gene and it is difficult to distinguish direct from indirect effects *in vivo*. Indirect effects on *Notch1 *gene expression are seen in *Pofut1 *[16] and *Mib1 *[17,18] null embryos defective in global signaling that exhibit increased *Notch1 *gene expression in the PSM, the forming somite and the forebrain at ~E8.75, suggesting that *Notch1 *expression at that stage is negatively regulated via signaling through Notch2, Notch3 and/or Notch4. By E9.5, *Notch1 *gene expression is inhibited in the absence of Pofut1 [16] or Mind bomb 1 [17,18]. Notch1 activation is also inhibited at E9.5 in embryos lacking RBP-Jκ [29].

The conditional *Notch1 *floxed allele allowed us to ask whether canonical Notch1 signaling is required for the generation of developmentally-competent eggs, or for fertilization, pre-implantation development, implantation or gastrulation. Previous experiments in which global Notch signaling was eliminated by the removal of Pofut1 or RBP-Jκ in oocytes suggest that Notch signaling is not required through any of the four mammalian Notch receptors until after gastrulation [20,21]. However, Pofut1 transfers fucose to EGF repeats with a consensus sequence that is found in a number of proteins including Notch ligands and Cripto [30,31]. While the presence of fucose on an EGF repeat is not required for the function of either Notch ligands [32] or Cripto [33], biological roles for *O*-fucose have only begun to be explored. In addition, recent experiments have shown that Notch lacking *O*-fucose may signal under certain circumstances [34,48]. Similarly, Notch-independent functions of RBP-Jκ have been described [35,36]. Finally, *Notch1 *is expressed in oocytes during oogenesis [37] and in ovulated eggs and developing blastocysts [11,12], leading to the prediction that Notch1 signaling must be important for pre-implantation development [11,12]. Thus it was important to examine this question directly. Our data clearly show that Notch1 signaling through canonical Notch ligands is in fact dispensable for oogenesis, ovulation, fertilization, blastogenesis, implantation and gastrulation (Fig. 4). They also show that expressing an inactive Notch receptor at the cell surface does not have an inhibitory effect on any of these developmental processes. However, non-canonical Notch1 signaling by a pathway yet to be discovered may be active in *Notch1*^*lbd*/*lbd *^oocytes or blastocysts. The fact that Notch1^lbd ^is well-expressed at the cell surface but not responsive to canonical Notch ligands means it may be used to search for novel Notch1 signaling pathways that may be active in pre-implantation development.

## Conclusion

In summary, we have shown that deletion of the mouse Notch1 ligand binding domain generates Notch1 of ~280 kDa that is well-expressed on the cell surface but cannot bind Delta1 nor be activated by Delta1 or Jagged1 in a co-culture signaling assay. Homozygous mutant embryos die at mid-gestation with defects similar to *Notch1 *null embryos. Oocyte-specific deletion of the ligand bindng domain does not impair oogenesis or development of maternal and zygotic embryos until after gastrulation.

## Methods

### Targeting of the Notch1 gene

To generate the Notch1 ligand binding domain deletion mutation *Notch1*^*lbd*^, an 1.6 kb region of genomic DNA containing exons 6 – 8 and two flanking sequences ~4.3 kb 5' (upstream) and ~2.9 kb 3' (downstream) were obtained by PCR from genomic DNA of WW6 ES cells [38] and cloned separately into pCR2.1 (T-vector, Invitrogen, Carlsbad, CA). A point mutation termed 12f was introduced into exon 8 by changing Thr466 to Ala [13]. The integrity of the three inserts was confirmed by DNA sequencing and they were subcloned between the three *loxP *sites in the pFlox vector [39] using *BamH*I, *Sal*I and *Xho*I with *Hind*III, respectively (Fig. 1A). The targeting vector was linearized using *Pvu*I (the *Pvu*I site between *Xho*I and *Hind*III in pFlox had been removed during subcloning). After gel purification, the plasmid was electroporated into WW6 ES cells using a Bio-Rad Gene Pulser (Bio-Rad, Hercules, CA) at 400 V and 250 μF. Following selection with 250 μg/ml active G418 (Invitrogen, Carlsbad, CA), resistant colonies were screened for homologous recombination by PCR using Takara Ex Taq (Takara Mirus Bio, Madison, WI) and primers N1ES-gF: 5'-GCTTCCCGCCTCCACTGTGCTATTGATGTTTG-3' from upstream of the 5' insertion site and pFlx-382R: 5'-GTTCCTCTTGCTGAACCACACTGCTCGATATTG-3' from the pFlox vector, and confirmed using primers pFlx-3521F: 5'-CTGTGCCTTCTAGTTGCCAGCCATCTGTTG-3' from the pFlox vector and DM142: 5'-CTGAAGCCTTCTCGGCAGGTGCATACGTAG-3' from downstream of the 3' insertion. Two positive ES clones were further characterized by Southern blot analysis after digestion by *Hind*III or *Hind*III and *EcoR*I. The probe P1415 is a genomic DNA fragment obtained by PCR from exons 14 to 15 of *Notch1 *using primers N1-ex14F: 5'-GTACAAGTGACTGTGCCCCTGGGTG-3' and N1-ex15R: 5'- CTGTATATGGCAGAGGACAGTTGCACTTG-3' and was used to determine integration into the endogenous *Notch1 *locus. Targeted ES cells were microinjected into C57Bl/6 blastocysts to obtain chimeric mice. Chimeras were crossed with transgenic mice carrying *Cre *recombinase under the control of a weak CMV promoter termed MeuCre40 [14] to obtain heterozygous floxed Notch1 (*Notch1*^+/*F*^) mice. Oocyte-specific Notch1 deletion was obtained by crossing *Notch1*^+/*F *^mice with transgenic mice carrying *Cre *recombinase under the control of the ZP3 promoter (ZP3*Cre*) as previously described [20,39]. The deletion mutation was confirmed by Southern blot analysis after *BamH*I digestion using probe P1415 downstream of the 3' insertion site. Genotyping was performed from tail DNA or yolk sac DNA using forward primer 5F: 5'-GTATGTATATGGGACTTGTAGGCAG-3', and reverse primer 6R: 5'-CTATGAGGGGTCACAGGACCAT-3' that generate a 363 bp product from the wild type Notch1 allele and a 466 bp product from the floxed *Notch1 *allele, or primers 5F and 9R: 5'-CTTCATAACCTGTGGACGGGAG-3' that generate a 575 bp product from the Notch1 ligand binding domain deletion allele. ZP3*Cre *transgenic mice were genotyped as described [39].

### Embryonic Stem Cell Isolation

Embryonic stem (ES) cell lines C1 (*Notch1*^+/+^), C2 (*Notch1*^+/*lbd*^) and A2 (*Notch1*^*lbd*/*lbd*^) were isolated from E3.5 blastocysts obtained from *Notch*^+/*lbd *^X *Notch*^+/*lbd *^crosses as described [40] and genotyped by PCR from yolk sac DNA. Other ES cell lines used were WW6 [38] (*Notch1*^+/+^), and 290-2 Notch1 null cells [10] termed *Notch1*^*in*32/*in*32 ^and kindly provided by Dr. G. D. Longmore. ES cells were routinely cultured on a STO SNL2 feeder cell layer [41] in ES medium (Knockout-DMEM supplemented with 15% fetal bovine serum; Gemini, West Sacramento, CA, 1 × non-essential amino acids, 1 × L-glutamine, 1000 U/ml ESGRO^® ^(Chemicon, Temecula, CA), 50 μM ί-mercaptoethanol, 25 mM HEPES, penicillin (50 U/ml) and streptomycin (50 μg/ml). All reagents were ES-qualified and from SpecialtyMedia (Phillipsburg, NJ) except where mentioned). To remove feeder cells, ES cells were passaged on gelatinized plates for 2 – 3 generations at an 1:10 ratio. For growth curves, ES cells were plated on 24-well plates at 5 × 10^4 ^cells per well and incubated at 37°C in an incubater with 5% CO_2_. Cells from triplicate wells were trypsinized and counted after 24, 48, 72 and 96 h using a Z1 Coulter particle counter (Beckman Coulter, Fullerton, CA). Cells from each well were counted 3 times.

### RT-PCR Analysis

Total RNA was isolated from ES cells using TRIZOL^® ^(Invitrogen, Carlsbad, CA) followed by DNase I (Promega, Madison, WI) digestion according to the manufacturer's instructions. cDNA was prepared using the Takara RNA PCR Kit ver 3.0 (Takara Mirus Bio, Madison, WI). RT-PCR analysis was performed using Notch1 forward primer: 5'-GCCCTTTGAGTCTTCATACATCTG-3' and reverse primer: 5'-GACATTGGAACTCATTGATCTTGT-3'. PCR products (500 bp from *Notch1*^*lbd *^and 1076 bp from *Notch1*^+^) were separated by agarose gel electrophoresis and visualized by ethidium bromide staining. *Gapdh *was used as control (forward: 5'-ACCACAGTCCATGCCATCAC-3'; reverse: 5'-TCCACCACCCTGTTGCTGTA-3', product size: 452 bp).

### Immunoblot Analysis

ES cells growing on gelatinized plates were washed with PBS and lysed in RIPA buffer (Upstate, Lake Placid, NY) containing the complete protease inhibitors 'cocktail' (Roche, Basel, Switzerland). After incubation for 30 min on ice, the lysate was microfuged and protein concentration of the supernate was determined by Bio-Rad D_*c *_protein assay (Bio-Rad, Hercules, CA). Lysates were resolved by 4–20% gradient SDS-PAGE, transferred to polyvinyldifluoride (PVDF) membranes and probed with Notch1 ECD antibody 8G10 (1:500; Upstate, Lake Placid, NY) to detect full length Notch1, or Notch1 antibody Val1744 (1:1000; Cell Signaling Technology, Beverly, MA) to detect activated Notch1. Horseradish peroxidase (HRP)-conjugated secondary antibodies were used to detect reactive bands visualized using Enhanced Chemiluminescence Reagent (Amersham Pharmacia Biotech, Piscataway, NJ). A β-Tubulin-III specific antibody (1:500; Sigma, St. Louis, MO) was used for loading control.

### Flow Cytometry

For cell surface Notch1 detection, ES cells growing on gelatinized plates at 70–80% confluence were dissociated using phosphate buffered saline (PBS) -based enzyme-free dissociation solution (SpecialtyMedia, Lavellette, NJ) for 10 min at 37°C. After washing with 10 ml medium, 5 × 10^5 ^ES cells were incubated with 1 μg anti-Notch1 antibody (8G10, Upstate, Lake Placid, NY) in 100 μl FACS binding buffer (Hank's balanced salt solution (HBSS) containing 3% BSA, 0.05% sodium azide, and 1 mM Ca^2+^) for 1 h at 4°C in the dark, followed after washing with 1 ml FACS binding buffer, by incubation with 1:100 Alexa488-conjugated anti-hamster IgG antibody (Invitrogen, Carlsbad, CA) in 100 μl FACS binding buffer. After washing with 1 ml FACS binding buffer, the cells were suspended into 400 μl FACS binding buffer. Dead cells were excluded by staining with 7-AAD (BD Pharmingen, San Diego, CA). Flow cytometry was performed on a FACS Calibur flow cytometer (BD Biosciences, San Diego, CA). Data files were analyzed using Flowjo software (Tree Star, San Carlos, CA).

### Notch Ligand Binding Assay

Soluble Notch ligand Delta1 with a human Fc tag (Delta1-Fc) [42,43] was kindly provided by Dr. Gerry Weinmaster. HEK-293T cells expressing Delta1-Fc were cultured in αMEM (Invitrogen) containing 10% fetal bovine serum (Gemini). At 70–80% confluence, the medium was changed to 293 SFM II serum-free medium (Invitrogen) and culturing was continued. After 3 days, conditioned medium was collected, cellular debris removed by centrifugation, and the supernatant stored at 4°C. The concentration of ligand was determined by comparison with known concentrations of human IgG antibody (Jackson Immunoresearch, West Grove, PA) detected by chemiluminescence (Amersham Pharmacia Biotech, Piscataway, NJ) after western blotting. For the binding assay, 70–80% confluent ES cells were dissociated from plates using PBS-based enzyme-free dissociation solution (SpecialtyMedia, Lavellette, NJ) for 10 min at 37°C. After washing with 10 ml medium, the single cell suspension of ES cells (5 × 10^5 ^cells) was incubated with 2 μg/ml Delta1-Fc in binding buffer (HBSS containing 3% BSA, 0.05% sodium azide, and 1 mM Ca^2+^) for 1 h at 4°C, followed after washing by incubation with 1:100 phycoerythrin (PE)-conjugated anti-human Fc antibody (Jackson Immunoresearch, West Grove, PA) for 30 min at 4°C. To inhibit Delta1 binding, 5 mM EDTA was added to the binding buffer. Flow cytometry was performed on a FACS Calibur flow cytometer. Ligand binding ability was determined by mean fluorescence intensity (MFI) of primary and secondary antibody binding minus the MFI of secondary antibody binding alone.

### Co-culture Notch Signaling Assay

The co-culture Notch signaling assay was performed essentially as previously described [20]. In brief, duplicate cultures of ES cells (*Notch1*^+/+^,(C1) and *Notch1*^*lbd*/*lbd *^(A2)) on 6-well plates were co-transfected with the TP1-luciferase Notch reporter plasmid and a *Renilla *luciferase reporter (pRL-TK; Promega, Madison, WI) and empty vector using FuGENE 6 (Roche, Basel, Switzerland). At 16 h post-transfection, ES cells were overlaid with 1 × 10^6 ^Jagged1-expressing L cells or Delta1-expressing L cells or parental L cells. At ~40 h post-transfection, firefly and *Renilla *luciferase activities were quantitated in cell lysates using a dual luciferase assay kit (Promega, Madison, WI) on Autolumat Plus LB 953 (Berthold Technologies, Bad Wildbad, Germany) according to the manufacturer's instructions. Ligand-dependent Notch activation is expressed as fold-induction of normalized firefly luciferase activity obtained from Notch ligand versus L cell co-cultures. Co-culture assays with *Notch1*^*in*32/*in*32 ^ES cells (290-2) compared to *Notch1*^+/+ ^ES cells (C1) were performed by the same method in 12-well plates using Lipofectamine (Invitrogen) for transfection of plasmids.

### Whole Mount *in situ *Hybridization

Embryos of E8.75, E9.0 or E9.5 from *Notch1*^+/*lbd *^crosses were harvested and DNA was prepared from yolk sac for genotyping. Embryos for whole mount *in situ *hybridization were fixed in 4% formaldehyde in PBS overnight at 4°C. Whole-mount *in situ *hybridization was performed as previously described [16]. The hybridization probes used were: *Notch1 *full length, ~9.5 kb or ~4.7 kb [44]; *Uncx4.1 *~1.7 kb [45], *Hes5 *~1.3 kb [46]; and *Myogenin *~1.5 kb [47]. Stained embryos were photographed in PBS through a phototube on Leica Wild M3Z stereomicroscope (Leica-Microsystems, Heerburgg, Switzerland) using a Canon S40 digital camera (Canon USA Inc., Lake Success, NY).

## List of abbreviations

lbd: ligand binding domain; Pofut1: protein *O*-fucosyltransferase 1; ES: embryonic stem; EGF: epidermal growth factor-like; N1: Notch1.

## Competing interests

The authors declare that they have no competing interests.

## Authors' contributions

CG and PS conceived the project and designed the experiments. CG performed the experiments in Figures 1, 2 and 4 and Tables 1 and 2 and wrote the paper. TL did the *in situ *hybridization experiments (Figure 3 and text) and helped to write the paper. XH did co-culture Notch signaling assays. All authors interpreted data and wrote the paper. All authors read and approved the final manuscript.
